# Identification of a Potential MiRNA–mRNA Regulatory Network for Osteoporosis by Using Bioinformatics Methods: A Retrospective Study Based on the Gene Expression Omnibus Database

**DOI:** 10.3389/fendo.2022.844218

**Published:** 2022-05-10

**Authors:** Shi Lin, Jianjun Wu, Baixing Chen, Shaoshuo Li, Hongxing Huang

**Affiliations:** ^1^ The Third Clinical Medical College of Guangzhou University of Chinese Medicine, Guangzhou University of Chinese Medicine, Guangdong, China; ^2^ Department of Development and Regeneration, KU Leuven, University of Leuven, Leuven, Belgium; ^3^ Laboratory of New Techniques of Restoration and Reconstruction of Orthopedics and Traumatology, Nanjing University of Chinese Medicine, Jiangsu, China; ^4^ Department of Orthopaedics, The Third Affiliated Hospital of Guangzhou University of Chinese Medicine, Guangdong, China

**Keywords:** osteoporosis, miRNAs, WGCNA, bioinformatics analysis, miRNA–mRNA regulatory network

## Abstract

**Introduction:**

As a systemic skeletal dysfunction, osteoporosis (OP) is characterized by low bone mass, impairment of bone microstructure, and a high global morbidity rate. There is increasing evidence that microRNAs (miRNAs) are associated with the pathogenesis of OP. Weighted gene co-expression network analysis (WGCNA) is a systematic method for identifying clinically relevant genes involved in disease pathogenesis. However, the study of the miRNA–messenger RNA (mRNA) regulatory network in combination with WGCNA in OP is still lacking.

**Methods:**

The GSE93883 and GSE7158 microarray datasets were downloaded from the Gene Expression Omnibus (GEO) database. Differentially expressed miRNAs (DE-miRNAs) and differentially expressed genes (DEGs) were analyzed with the limma package. OP-related miRNAs from the most clinically relevant module were identified by the WGCNA method. The overlap of DE-miRNAs and OP-related miRNAs was identified as OP-related DE-miRNAs. Both upstream transcription factors and downstream targets of OP-related DE-miRNAs were predicted by FunRich. An intersection of predicted target genes and DEGs was confirmed as downstream target genes of OP-related DE-miRNAs. With the use of clusterProfiler in R, Gene Ontology (GO) annotation and Kyoto Encyclopedia of Genes and Genomes (KEGG) pathway enrichment were performed on target genes. Finally, both the protein–protein interaction (PPI) network and miRNA–mRNA network were constructed and analyzed.

**Results:**

A total of 79 OP-related DE-miRNAs were obtained, most of which were predicted to be regulated by specificity protein 1 (SP1). Subsequently, 197 downstream target genes were screened out. The target genes were enriched in multiple pathways, including signaling pathways closely related to the onset of OP, such as Ras, PI3K-Akt, and ErbB signaling pathways. Through the construction of the OP-related miRNA–mRNA regulatory network, a hub network that may play a prominent role in the formation of OP was documented.

**Conclusion:**

By using WGCNA, we constructed a potential OP-related miRNA–mRNA regulatory network, offering a novel perspective on miRNA regulatory mechanisms in OP.

## Introduction

As a systemic skeletal dysfunction, osteoporosis (OP) is characterized by low bone mass and impairment of bone microstructure, consequently compromising bone strength and increasing fracture risk (1, 2). During the progression of OP, patients often develop sarcopenia, which leads to frailty syndrome (3). Moreover, OP has a high morbidity rate among the global elderly, especially in postmenopausal women (1, 4). Due to its silent nature, patients suffering from OP often feel no symptoms until the first osteoporotic fracture occurs (1). Annually, over 8.9 million fractures are caused by OP worldwide, which means that an osteoporotic fracture happens every 3 s (5). Among osteoporotic fractures, the spine, hip, distal forearm, and proximal humerus fractures tend to be the most frequent (6, 7). Elderly patients suffering from osteoporotic fractures often require hospitalization, resulting in impaired quality of life, long-lasting medical care, disability, and even death (7, 8). This poses a high economic and social burden worldwide (9) and is a global public health challenge. Hence, a better understanding of the regulatory mechanisms of OP is of great importance, which is conducive to developing novel and effective therapies.

MicroRNAs (miRNAs), commonly 19–23 nt in length, are small non-coding RNAs that are highly conserved and negatively regulate gene expression by transcriptional inhibition through binding to 3′-untranslated regions (3′ UTRs) of target messenger RNAs (mRNAs) (10, 11). With the advances in research, ample evidence indicates that miRNAs regulate a wide range of processes, including proliferation, differentiation, apoptosis, and development (12, 13). Consequently, miRNAs play a crucial role in the pathogenesis of multiple conditions, including inflammation, metabolic disorders, and cancers (12, 14). Studies indicate that miRNAs can maintain the equilibrium of bone resorption and bone formation, a balance that is disturbed in OP. MiR-214-3p derived from osteoclasts inhibits bone formation both *in vivo* and *in vitro* by transferring to osteoblast (15). It is confirmed that elevated miR-214 levels are correlated with reduced bone formation in bone specimens from aged fractured patients, further demonstrating the inhibition of osteoblast proliferation *in vitro* by modulating ATF4 (16). A series of miRNAs were identified to regulate the osteogenic process and bone-forming activity (14, 17, 18). Although the effects of miRNAs on OP have been investigated through various studies, their molecular mechanisms remain to be clarified. Therefore, further in-depth studies of regulatory mechanisms of miRNAs in OP are needed, which may provide a comprehensive understanding of OP development. Meanwhile, potential biomarkers may be identified for OP diagnosis and treatment.

Recent developments in different algorithms and research strategies have contributed to identifying potential mechanisms of gene networks, enabling further understanding of many diseases (19–21). Weighted gene co-expression network analysis (WGCNA) is one of such tools and has been widely applied to identify disease-related biomarkers, such as inflammatory diseases, metabolic diseases, and cancers (22–25). It is a systematical bioinformatics technique for clustering biologically highly-relevant genes into modules, which also defines correlations between modules and clinical traits (25). These modules may contain critical genes that are valuable biomarkers or therapeutic targets for predicting, diagnosing, and treating a specific disease (21). This research conducted a bioinformatics analysis using OP-related transcriptomic profiles from the Gene Expression Omnibus (GEO) database. Key modules of OP-related miRNAs were screened by the WGCNA method. Subsequently, differentially expressed miRNAs (DE-miRNAs) and differentially expressed genes (DEGs) of OP were identified. Transcription factors (TFs) of OP-related DE-miRNAs were specified. Moreover, functional gene annotations were performed to investigate the relevant mechanisms in OP. In order to clarify the miRNA–mRNA regulatory mechanism in OP, we constructed a miRNA–mRNA regulatory network, from which a key subnetwork was identified.

## Materials and Methods

### Microarray Data Collection

**Figure 1:**
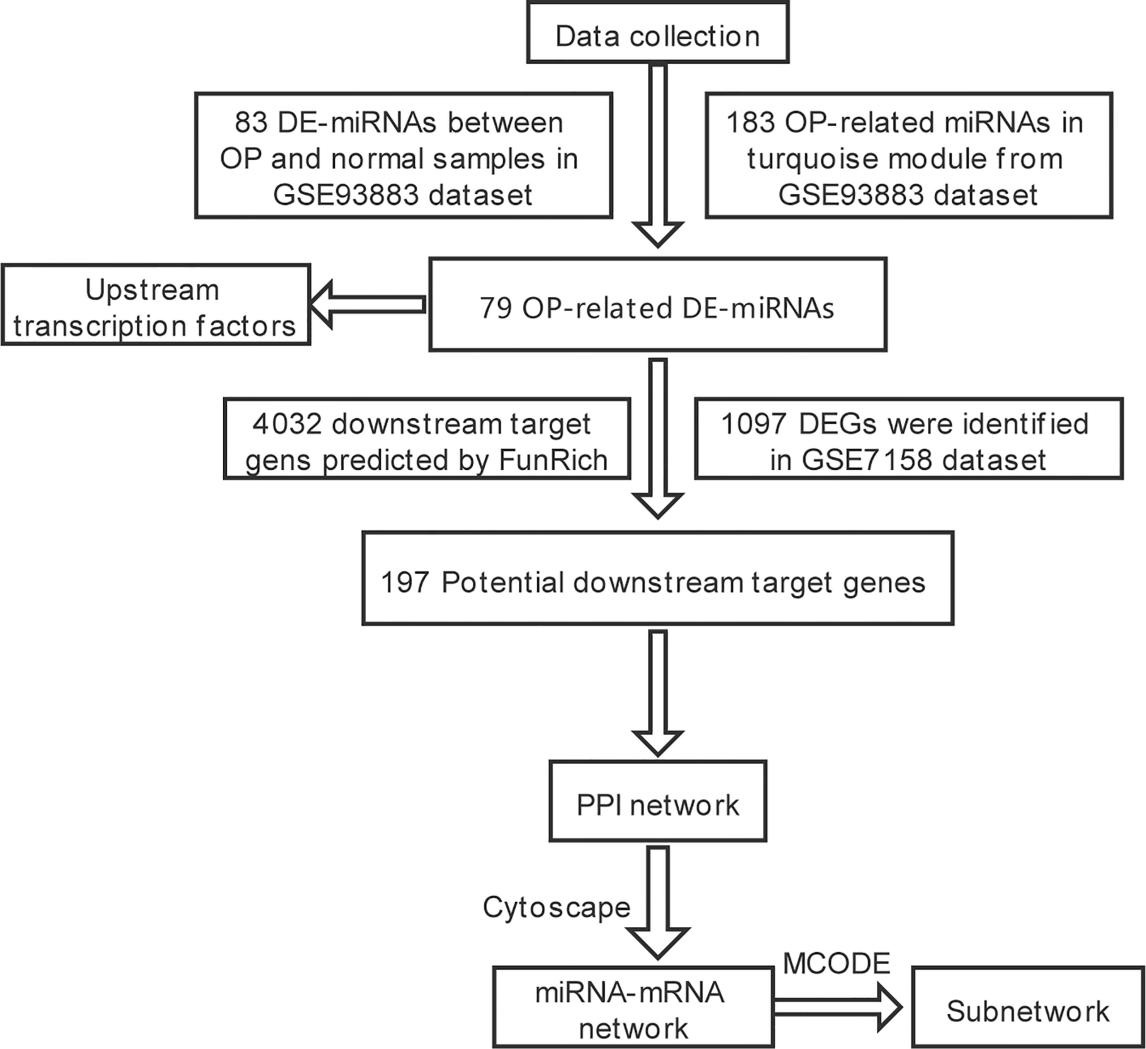
Workflow of research routine to construct the miRNA–mRNA regulatory network in OP. miRNA, microRNA; mRNA, messenger RNA; OP, osteoporosis; DE-miRNAs, differentially expressed miRNAs; DEGs, differentially expressed genes.

The study flowchart is shown in **Figure 1**. Data of miRNAs and mRNAs were acquired from GSE93883 and GSE7158 datasets, respectively. Both datasets were obtained from the GEO database (http://www.ncbi.nlm.nih.gov/geo/). The GSE93883 dataset contains 6 healthy control samples and 12 primary OP samples, using the microarray platform GPL18058. The GSE7158 dataset involved 14 high peak bone mass (PBM) and 12 low PBM participants (according to the hip Z-score), using the microarray platform GPL570. The raw data of the GSE93883 dataset were available in GFP format, while those of the GSE7158 dataset were in CEL format.

### Data Preprocessing, Differentially Expressed MiRNAs, and Differentially Expressed Genes Screening

The mRNA and miRNA expression matrix was obtained by using Perl script (www.perl.org/). DE-miRNA analysis between OP and normal samples and DEG analysis between low PBM and high PBM samples were performed utilizing the limma (Linear Models for Microarray Data) algorithm. The DEGs meet the criterion |logFC| > 2 and p-value <0.05, while the DE-miRNAs meet the criterion |logFC| > 2 and adjusted p-value <0.05.

### Identification of Osteoporosis-Related MiRNAs by Weighted Gene Co-Expression Network Analysis

A weighted gene co-expressed network of miRNAs using the “WGCNA” package in R generates a scale-free weighted gene co-expressed network (26). Co-expressed genes and modules related to clinical traits were identified. A thresholding power β value of 13 was determined for the clustering of data to detect outliers and produce a scale-free network. Subsequently, a hierarchical clustering tree was used to identify gene modules, and the average-linkage hierarchical clustering using topological overlap matrix-based dissimilarity measure was used to detect gene modules (27). MEDissThres was set to 0.25 to automatically combine similar modules. The correlation between modules and clinical traits was confirmed using Pearson’s correlation coefficient. Finally, OP-related DE-miRNAs were obtained by overlapping the DE-miRNAs and miRNAs of the most OP-related module.

### Screening for Upstream Transcription Factors

The current study screened potential transcription factors of OP-related DE-miRNAs by using FunRich (version 3.1.3) software (http://www.funrich.org), enabling the identification of enriched transcription factors depending on the gene set (28).

### Potential Downstream Target Genes

In this study, putative targets genes of OP-related DE-miRNAs were predicted by FunRich. Next, an overlap of predicted target genes and DEGs from the GSE7158 dataset was taken, identifying the potential targets of OP-related DE-miRNAs.

### Gene Ontology and Pathways Analysis

Gene Ontology (GO) and Kyoto Encyclopedia of Genes and Genomes (KEGG) pathway analysis were performed *via* the “clusterProfiler” package in R to determine the biological function of potential downstream target genes of OP-related DE-miRNAs. A p-value <0.05 was considered statistically significant.

### Construction of Protein–Protein Interaction Network

The protein–protein interaction (PPI) pairs between potential downstream target mRNAs of OP-related DE-miRNAs were identified *via* the String (Search Tool for Retrieval of Interacting Genes; http://string-ab.org/) database, applying high confidence >0.700. Furthermore, the disconnected mRNAs were removed from the PPI network.

### Construction and Analysis of MiRNA–mRNA Network

The interactions between OP-related DE-miRNAs and mRNAs from the PPI network were obtained. An OP-related miRNA–mRNA network was then constructed by using Cytoscape v3.7.1. A subnetwork was screened by the MCODE algorithm, which was considered the hub miRNA–mRNA regulatory network of OP.

## Results

### Identification of Significant Osteoporosis-Related Gene Module

**Figure 2:**
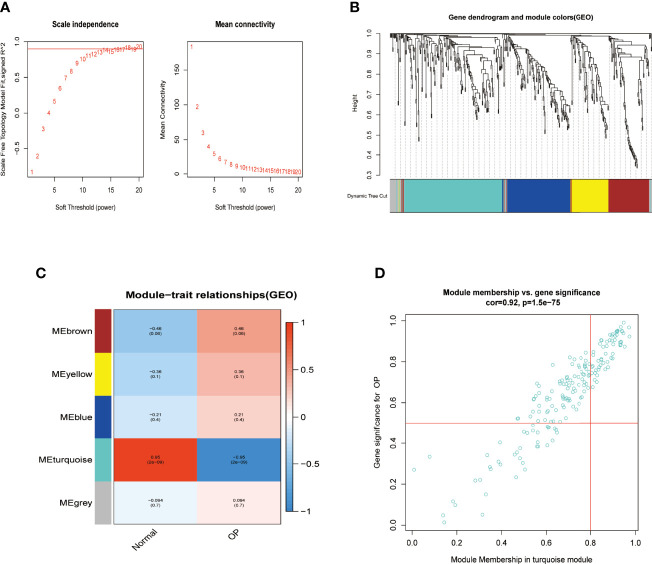
Identifying significant gene modules associated with OP. **(A)** β = 13 was selected to establish a scale-free network in the GSE93883 dataset. **(B)** Gene clustering based on topological dissimilarity and module colors in GSE93883 dataset. **(C)** Correspondence between gene modules with osteoporosis participants or normal ones among the GSE93883 dataset. **(D)** The correlation between gene significance and module membership in the GSE93883 dataset was plotted. OP, osteoporosis.

OP samples of miRNAs from the GSE93883 dataset were used for weight gene co-expression network construction, and the pickSoftThreshold function in the WGCNA package was used to determine the value of soft thresholding power β. In the present study, a β value of 13 combined with scale independence reached 0.9, and a high level of mean connectivity was observed (**Figure 2A**). The turquoise module (183 miRNAs) was one of the five clustered co-expressed gene modules and was the most relevant to OP (R = −0.95, p-value ≪ 0.05, **Figures 2B, C**). Furthermore, a strong correlation was detected between the turquoise module and its miRNAs (R = 0.92, p-value ≪ 0.05, **Figure 2D**).

### Screening of Differentially Expressed MiRNAs, Differentially Expressed Genes, and Osteoporosis-Related Differentially Expressed MiRNAs

**Figure 3:**
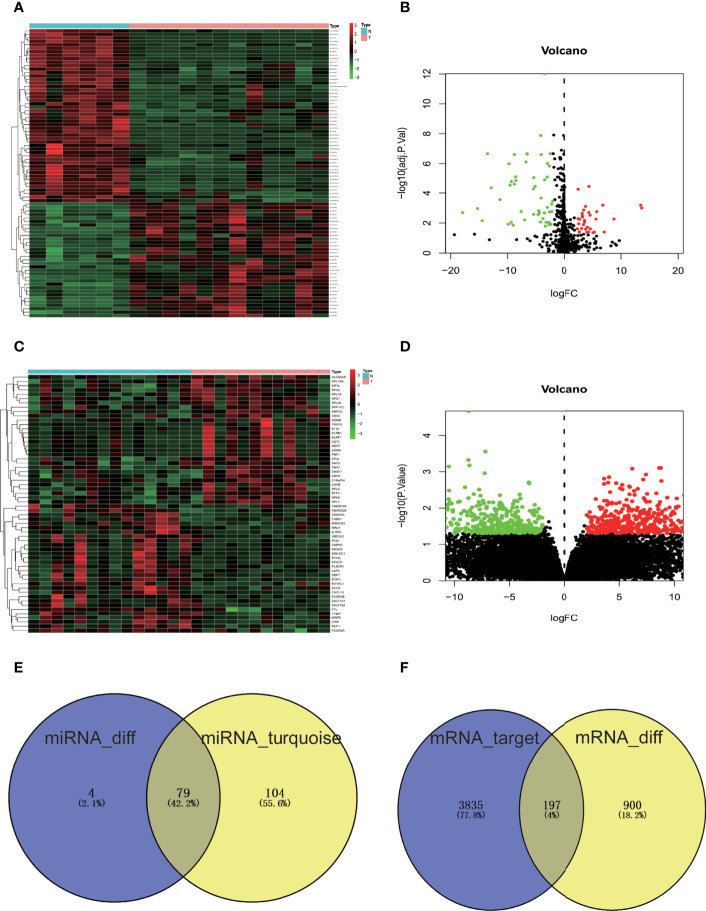
Screening of DE-miRNAs, DEGs, and OP-related DE-miRNAs. **(A)** Heatmap of miRNA expression in GSE93883 dataset. **(B)** GSE93883 miRNA expression volcano plot. **(C)** Gene expression heatmap of GSE7158 dataset. **(D)** GSE7158 gene expression volcano plot. Black color indicates non-significant genes, while red/green color represents upregulated/downregulated DE-miRNAs or DEGs. **(E)** Venn diagram of DE-miRNAs and miRNAs closely related to OP in the turquoise module. **(F)** Venn diagram of predicted targets of OP-related DE-miRNAs and DEGs of GSE7158 dataset. The number of miRNAs or genes in each group is displayed in Venn diagrams. N, normal group; T, osteoporosis group; OP, osteoporosis; miRNA, microRNA; mRNA, messenger RNA; DE-miRNAs, differentially expressed miRNAs; DEGs, differentially expressed genes.

DE-miRNAs in the GSE93883 dataset and DEGs in the GSE7158 dataset were screened *via* the “limma” package in R. The screening results revealed 83 DE-miRNAs, of which 33 were upregulated and 50 were downregulated, meeting the |logFC| > 2 and adjusted p-value <0.05 criterion (**Figures 3A, B**). As a result, 1,097 DEGs in the GSE7158 dataset were screened, fitting the |logFC| > 2, and p-value <0.05 criteria. Among the DEGs, 623 were upregulated and 474 were downregulated (**Figures 3C, D**). Subsequently, 79 OP-related DE-miRNAs were obtained by overlapping DE-miRNAs and miRNAs in the turquoise module (**Figure 3E**).

### Upstream Transcription Factors for Osteoporosis-Related Differentially Expressed MiRNAs

**Figure 4:**
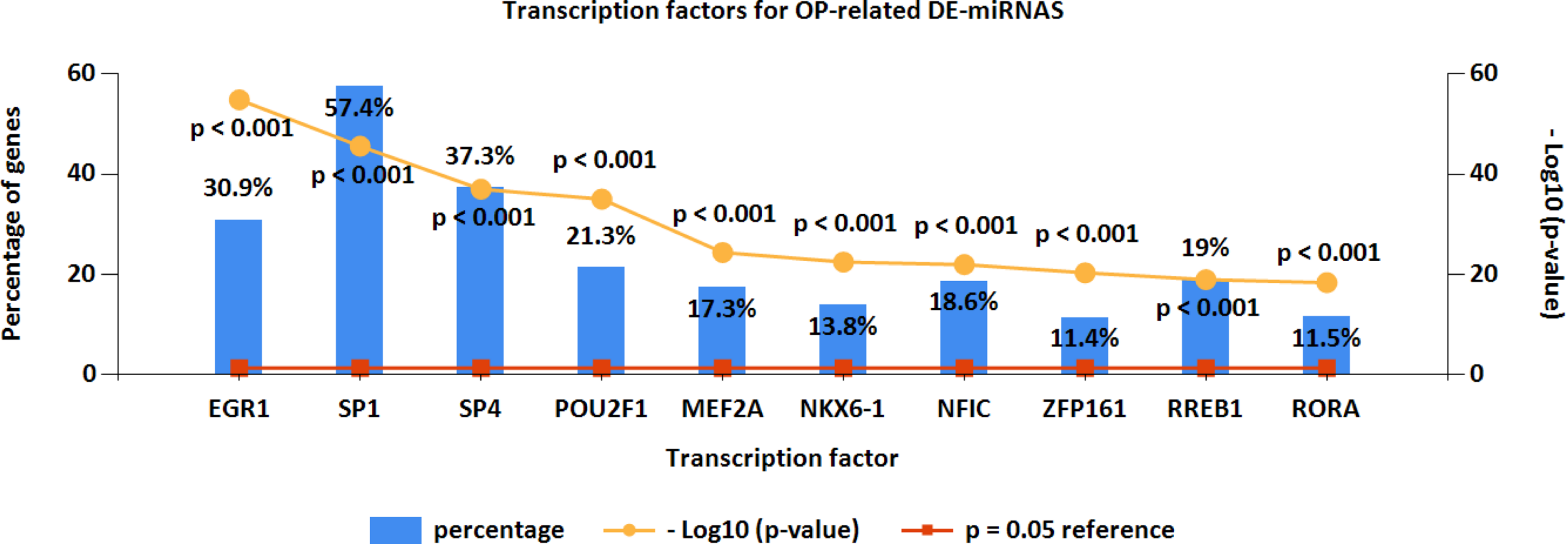
Upstream transcription factors for OP-related DE-miRNAs. OP, osteoporosis; DE-miRNAs, differentially expressed miRNAs.

Furthermore, the upstream transcription factors for OP-related DE-miRNAs were predicted by FunRich (**Figure 4**). The most significant transcription factors were EGR1, SP1, SP4, POU2F1, MEF2A, NKX6-1, NFIC, ZFP161, RREB1, and RORA.

### Putative Downstream Targets of Osteoporosis-Related Differentially Expressed MiRNAs

A total of 4,032 genes targeted by OP-related DE-miRNAs were identified by FunRich (**Figure 3F**). In order to refine the results, an intersection (197 genes, **Figure 3F**) of the predicted target genes and DEGs was obtained, identifying the potential downstream target genes of OP-related DE-miRNAs.

### Gene Ontology and Kyoto Encyclopedia of Genes and Genomes Enrichment

**Figure 5:**
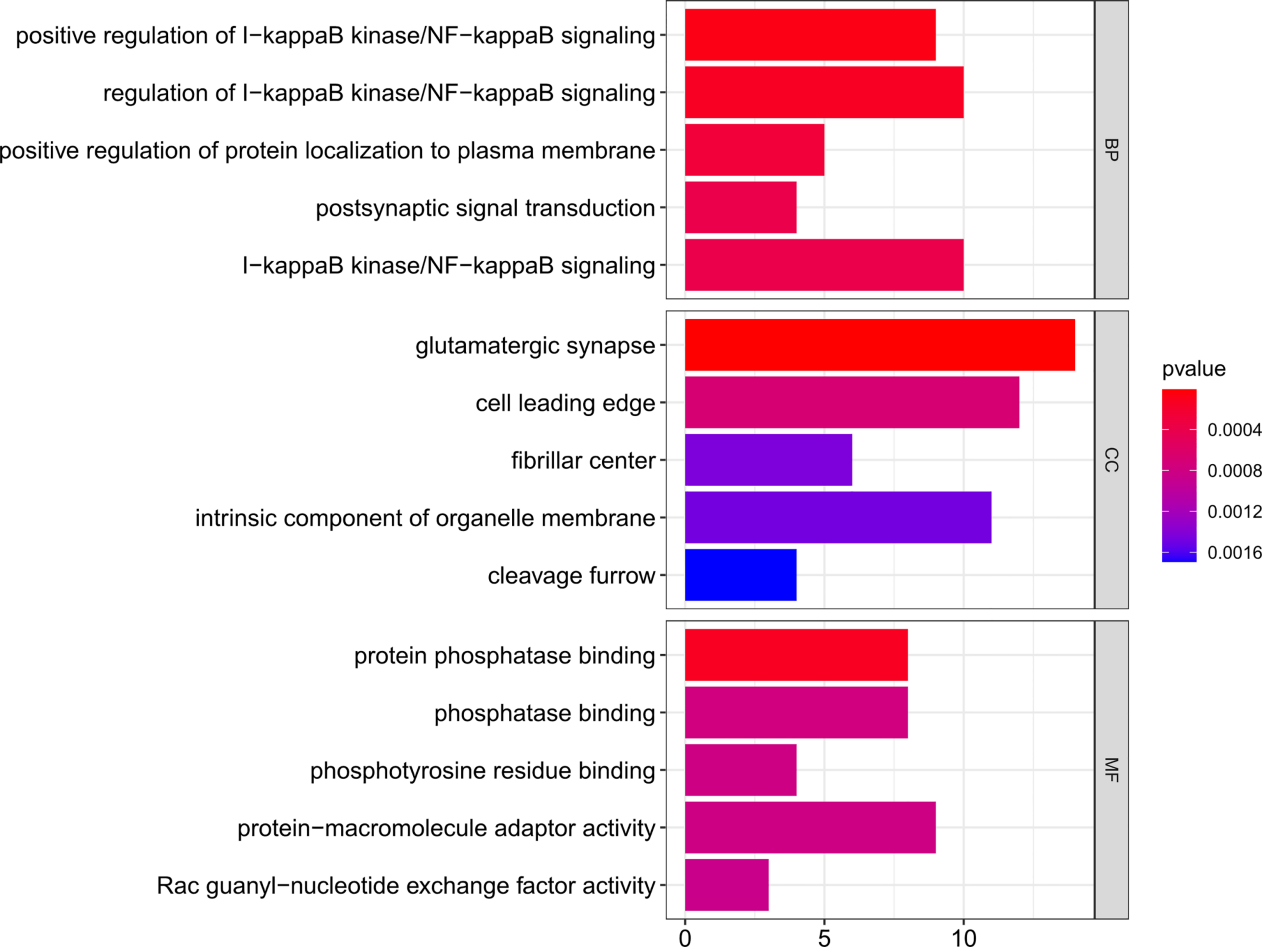
GO functional enrichment of downstream targets of OP-related DE-miRNAs. The x-axis shows enriched gene numbers and the color represents significance. GO terms are shown on the y-axis. p < 0.05. OP, osteoporosis; DE-miRNAs, differentially expressed miRNAs; DEGs, differentially expressed genes; GO, Gene Ontology; KEGG, Kyoto Encyclopedia of Genes and Genomes; BP, biological process; CC, cellular component; MF, molecular function.

Then, using clusterProfiler in R, GO functional and KEGG enrichment analyses were performed on the downstream target genes to further understand their biological functions. As represented in **Figure 5**, biological process (BP) was significantly enriched in positive regulation of I-kappaB kinase/NF-κB signaling, positive regulation of protein localization to the plasma membrane, and postsynaptic signal transduction; cellular component (CC) was particularly enriched in glutamatergic synapse, cell leading edge, and fibrillar center; molecular function (MF) was mainly enriched in protein phosphatase binding, phosphatase binding, and phosphotyrosine residue binding.

**Figure 6:**
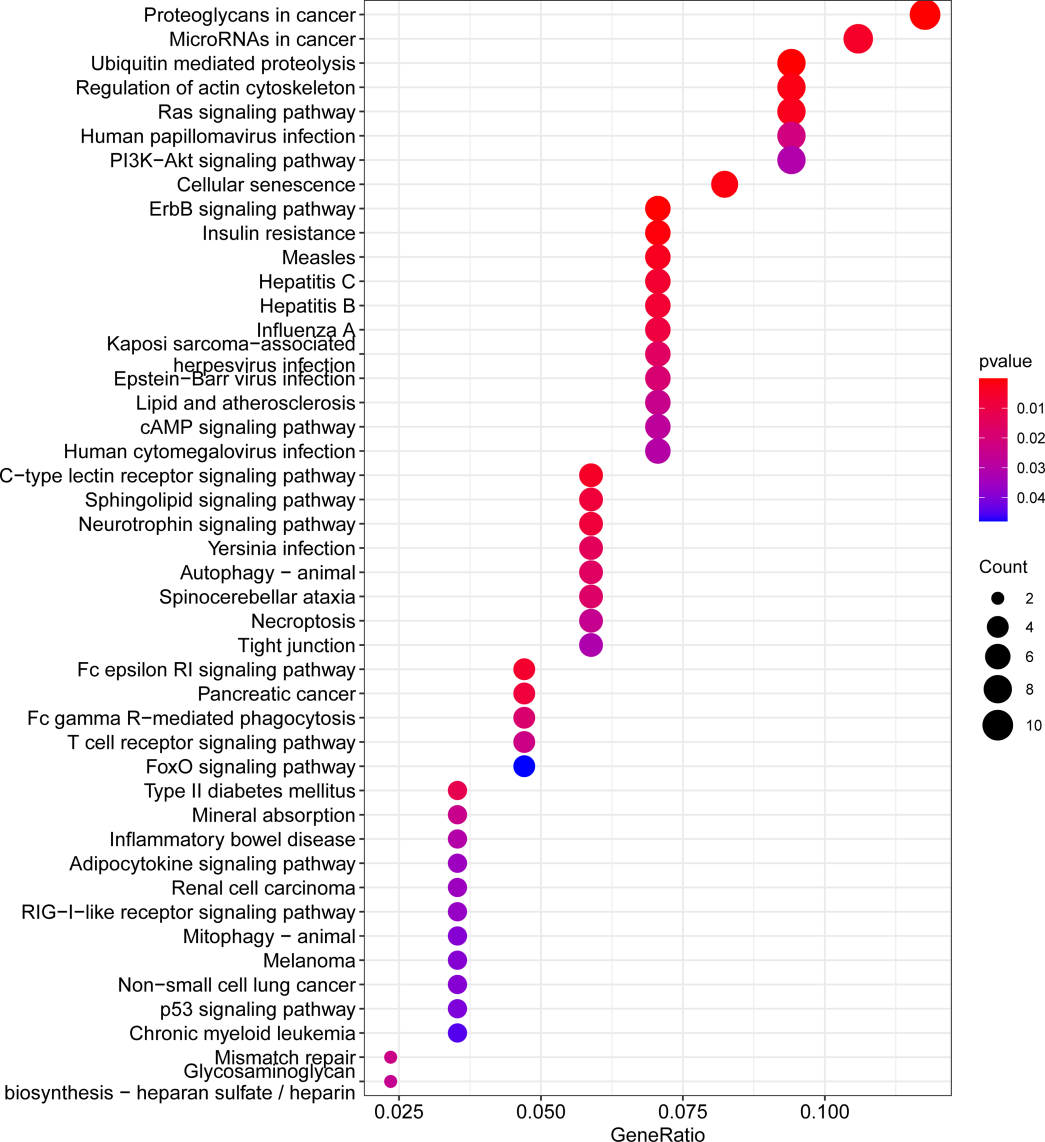
KEGG pathway enrichment for downstream target genes of OP-related DE-miRNAs. The enriched gene ratio is shown on the x-axis, color represents significance, and the pathway terms are displayed on the y-axis. p < 0.05. OP, osteoporosis; DE-miRNAs, differentially expressed miRNAs; KEGG, Kyoto Encyclopedia of Genes and Genomes.

Furthermore, the KEGG pathway analysis of the 197 downstream target genes is shown in **Figure 6**. Among all the pathways enriched, the top 10 most significant pathways were as follows: proteoglycans in cancer, miRNAs in cancer, ubiquitin-mediated proteolysis, regulation of actin cytoskeleton, Ras signaling pathway, human papillomavirus infection, PI3K-Akt signaling pathway, cellular senescence, ErbB signaling pathway, and insulin resistance (**Figure 6**). Notably, Ras, PI3K-Akt, and ErbB signaling pathway were closely related to the onset of OP.

### Protein–Protein Interaction and MiRNA–mRNA Network

**Figure 7:**
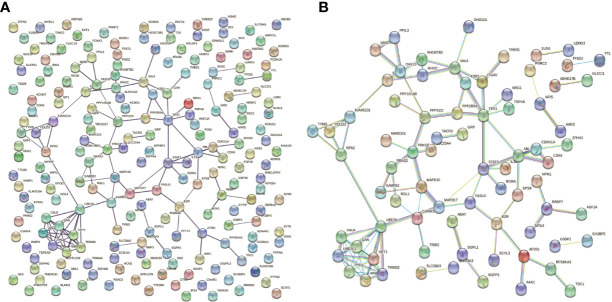
PPI network of downstream targets of OP-related DE-miRNAs. miRNA, microRNA; PPI, protein–protein interaction; OP, osteoporosis; DE-miRNAs, differentially expressed miRNAs.

**Figure 8:**
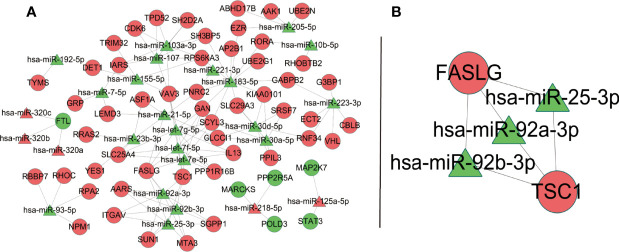
OP-relevant miRNA–mRNA regulatory network. Ellipse represents target genes of miRNAs; triangle represents miRNAs. Red dots represent miRNAs or targets that are upregulated, while green dots represent those that are downregulated. **(A)** MiRNA–mRNA regulatory network of OP. **(B)** A subnetwork identified by the MCODE algorithm in Cytoscape. OP, osteoporosis; miRNA, microRNA; mRNA, messenger RNA.

The PPI network of downstream targets of OP-related DE-miRNAs was constructed by the STRING online database with a high confidence >0.700 applied (**Figure 7A**). The disconnected nodes (genes) were removed from the PPI network (**Figure 7B**). The interactions between OP-related DE-miRNAs and genes from **Figure 7B** were obtained. A miRNA–mRNA network was then constructed by using Cytoscape (**Figure 8A**), from which the hub miRNA–mRNA regulatory network of OP was identified by the MCODE algorithm (**Figure 8B**), comprising three downregulated miRNAs (has-miR-25-3p, has-miR-92a-3p, and hsa-miR-92b-3p) and two upregulated mRNAs (FASLG and TSC1).

## Discussion

Over the past decade, a growing number of studies have focused on OP diagnosis and treatment (29). Patients suffering from OP usually receive treatment based on a lifestyle approach and a precise assessment of the risk of fracture (30–32). Moreover, patients at high risk were treated with pharmacological approaches including bisphosphonates, teriparatide, denosumab, and recently anti-sclerostin antibodies (33). However, concerns about rare side effects of pharmacological treatment, particularly bisphosphonates, and the absence of clear evidence in support of their long-term efficacy are leading many patients who could benefit from drug therapy to not take these drugs (29), indicating a limited understanding of OP pathogenesis. Therefore, a better understanding of the regulatory mechanism of OP is required to develop new treatments or improve existing therapies for OP. Advances in bioinformatics have facilitated the identification of genetic changes in disease development. The current study detected five modules in GSE93883 data, with the turquoise module being the most correlated with OP. Comparing the DE-miRNAs with miRNAs in the turquoise module (**Figure 3E**), 79 shared miRNAs were identified for further analysis.

In recent years, a growing number of miRNAs have been reported to be involved in diverse biogenesis pathways by mediating gene silencing (11). To better understand the functions of 79 OP-related DE-miRNAs, their downstream target genes were predicted by FunRich. Furthermore, by comparing the predicted genes to DEGs in the GSE7158 dataset, 197 shared genes were screened as downstream targets of OP-related DE-miRNAs (**Figure 3F**). These 197 genes were enriched in a range of functions and pathways based on the GO and KEGG analyses. Evidence suggests that these GO terms and pathways are important in OP. For example, in a high-content imaging-based *in vivo* chemical screen, NF-κB signaling was identified as a cell-autonomous inhibitor of osteoblast differentiation (34). Ras is a crucial regulator of normal bone growth and development (35). During intramembranous osteogenesis induced by tensile force, Ras was proven to promote chemotaxis of chemotaxis in preosteoblasts both *in vivo* and *in vitro* (36). Fisher et al. (37) identified the requirement for ErbB signaling to maintain osteoblast proliferation involved in the timely progression of periosteal osteoblast differentiation. Evidence showed that apoptosis of osteoblasts in OP mice induced by ovariectomy was induced by blockade of the PI3K/AKT signaling (38), while activation of PI3K/AKT signaling pathway facilitated Runx2 and osterix expression in osteoblast (39). In combination with the above evidence, the 197 genes may be involved in the pathogenesis of OP.

Transcription factors can modulate miRNA expression (40). Therefore, transcription factor prediction was performed using FunRich to identify potential transcription factors regulating the OP-related DE-miRNAs. As a result, specificity protein 1 (SP1) accounted for the highest percentage of OP-related DE-miRNAs. The C2H2-type zinc-finger transcription factor SP1 has been extensively documented in OP and regulates cell growth, differentiation, apoptosis, and others (41). There is a close relationship between SP1 binding site polymorphism at COL1A1 gene and OP risk among men and women (42). SP1 can also stimulate specific miRNA to inhibit osteogenesis *via* modulating the LRP5/Wnt/β-catenin pathway (43).

The potential regulatory mechanisms of OP were explored by constructing the miRNA–mRNA network of OP, providing further insight into OP. A hub miRNA–mRNA regulatory network, comprising three downregulated miRNAs (has-miR-25-3p, has-miR-92a-3p, and hsa-miR-92b-3p) and two upregulated mRNAs (FASLG and TSC1), was identified. MiR-25-3p can promote osteoclast activity and could be a powerful target for handling skeletal disorders characterized by reduced bone formation (44). TSC1 is a key regulator of mTORC1, and in a RANKL-induced OP mouse model, deletion of Tsc1 in osteoclast lineage cells prevented bone resorption (45). Younes et al. (46) identified FASLG as one of the genetic risk factors that contributed to low bone density in the Qatari population, based on the whole genomes sequenced from 3,000 individuals from the Qatar Biobank. There was no direct evidence that miR-92a-3p and miR-92b-3p were correlated to OP. However, studies have shown that they contributed to different diseases involving different pathways (47–50). Indeed, we provided a reference for OP therapeutic targets, especially those in the hub network. We believe that it may promote the development of new treatments to some extent.

A potential OP-related miRNA–mRNA regulatory network was identified by analyzing microarray profile data and taking clinical traits into consideration using the WGCNA algorithm, providing new insight into OP in terms of biological functions and potential pathophysiological mechanisms. However, the present study has several limitations. The sample size of each GEO dataset in our study was relatively small, and the OP-related miRNA–mRNA interactions were only predicted by public databases, which need further validation through *in vivo* and *in vitro* experiments. Moreover, most proposed pathways (RAS and ERB) are also involved in tumorigenesis, and evidence is only from *in vitro* studies, which limits the results of the present study and the possibility of an intervention. Finally, our study was limited to primary OP.

## Conclusion

Overall, we established a potential OP-related miRNA–mRNA regulatory network *via* the WGCNA method, offering a novel insight into the regulatory mechanisms of miRNAs in the development of OP. Several key miRNAs and mRNAs, including has-miR-25-3p, has-miR-92a-3p, hsa-miR-92b-3p, FASLG, and TSC1, were identified from the network and may act as key biomarkers or clinical targets for OP. We sincerely hope that our efforts could contribute to future in-depth investigations into OP.

## Data Availability Statement

Publicly available datasets were analyzed in this study. This data can be found here: https://www.ncbi.nlm.nih.gov/, GSE93883; GSE7158.

## Author Contributions

JW and SHL performed the literature search, and HH conceived and designed the project. SL and BC performed the data analysis. SL wrote the paper. HH reviewed and amended the manuscript. The manuscript has been read and approved by all authors.

## Funding

This work was supported by the National Natural Science Foundation of China (Grant No. 81973886), by Guangzhou University of Chinese Medicine “Double first-class” and High-level University Discipline Collaborative Innovation Team Project (Grant No. 2021XK21), and by the Postgraduate Research and Innovation Project of Guangzhou University of Chinese Medicine in 2021 (Doctoral candidate: Shi Lin).

## Conflict of Interest

The authors declare that the research was conducted in the absence of any commercial or financial relationships that could be construed as a potential conflict of interest.

## Publisher’s Note

All claims expressed in this article are solely those of the authors and do not necessarily represent those of their affiliated organizations, or those of the publisher, the editors and the reviewers. Any product that may be evaluated in this article, or claim that may be made by its manufacturer, is not guaranteed or endorsed by the publisher.

## Abbreviations

Nt, nucleotides; PBM, peak bone mass; EGR1, early growth response 1; SP1, specificity protein 1; SP4, specificity protein 4; POU2F1, POU domain class 2 transcription factor 1; MEF2A, myocyte enhancer factor 2A; NKX6-1, NK6 homeobox 1; NFIC, nuclear factor I-C; ZFP161, zinc finger protein 161; RREB1, Ras-responsive element-binding protein 1; RORA, RAR-related orphan receptor A; FASLG, FAS/FAS ligand; TSC1, tuberous sclerosis complex 1; GO, Gene Ontology; KEGG, Kyoto Encyclopedia of Genes and Genomes; BP, biological process; CC, cellular component; MF, molecular function.
